# Supplementary material for: Translation and validation of the Bahasa Malaysia version of the Nasal Obstruction Symptom Evaluation scale (M-NOSE)
Source: PeerJ. 2024 Aug 1;12:e17825. doi: 10.7717/peerj.17825 (PMC11298164; doi:10.7717/peerj.17825)
Supplement: Supplemental Information 1 [file peerj-12-17825-s001.pdf]

Supplementary Table S1. The Malay version of the Nasal Obstruction Symptom Evaluation Scale (M-NOSE).

Nasal Obstruction Symptom Evaluation Scale (NOSE)

Skala Penilaian Gejala Hidung Tersumbat (M-NOSE)

Over the past **1 month**, how much of a **problem** were the following conditions for you?

Please **circle** the most correct response

*Dalam tempoh **1 bulan** yang lalu, banyak mana keadaan ini menjadi **masalah** untuk anda.*

*Sila **bulatkan** maklum balas yang paling tepat.*

	Not a problem<br>*Tiada masalah*	Very mild problem<br>*Masalah yang ringan*	Moderate problem<br>*Masalah yang sederhana*	Fairly bad problem<br>*Masalah yang teruk*	Severe problem<br>*Masalah yang sangat teruk*	Score<br>*Skor*
Nasal congestion or stuffiness<br>*Hidung ketat*	0	1	2	3	4	
Nasal blockage or obstruction<br>*Hidung tersumbat*	0	1	2	3	4	
Trouble breathing through nose<br>*Susah bernafas melalui hidung*	0	1	2	3	4	
Trouble sleeping<br>*Tidur terganggu*	0	1	2	3	4	
Unable to get enough air through nose during exercise or exertion<br>*Pengudaraan melalui hidung tidak mencukupi ketika melakukan senaman atau aktiviti fizikal*	0	1	2	3	4	
					Total score<br>*Jumlah skor*	__ x 5 = __
