# Supplementary material for: Translation and validation of the Bahasa Malaysia version of the Nasal Obstruction Symptom Evaluation scale (M-NOSE)
Source: PeerJ. 2024 Aug 1;12:e17825. doi: 10.7717/peerj.17825 (PMC11298164; doi:10.7717/peerj.17825)
Supplement: Supplemental Information 4 [file peerj-12-17825-s004.pdf]

The NOSE Scale © 2003, the American Academy of Otolaryngology–Head and Neck Surgery Foundation.)

© 2024 American Academy of Otolaryngology—Head and Neck Surgery Foundation

Contact Us

1650 Diagonal Rd

Alexandria VA 22314

1-703-836-4444

Webpage: https://www.entnet.org/resource/facial-plastics-rhinology-outcome-tool-nose-scale/

The NOSE scale is free to use, as indicated on the website, but you must cite the original study where it was published.

Facial Plastics/Rhinology Outcome Tool: NOSE Scale

APRIL 16, 2021

Description

Disease-specific health status instrument for use in patients with nasal obstruction

Targeted Age

Adults

Items (Domains)

5 (N/A)

Estimated Completion Time

5 minutes

Reliability/Validity Assessment

Yes/Yes

This study was funded by the AAO-HNS/F. If others wish to use the tool, there is no cost, we simply ask that you reference the study.

Literature

1) Stewart MG, Witsell DL, Smith TL, Weaver EM, Yueh B, Hannley MT. Development and validation of the Nasal Obstruction Symptom Evaluation (NOSE) scale. Otolaryngol Head Neck Surg 2004;130:157-63.

Related Content

Rhinology Outcome Tool: RSOM-31
RESEARCH OUTCOME TOOLS

Rhinology Outcome Tool: RSUI
RESEARCH OUTCOME TOOLS

Rhinology Outcome Tool: SN-[illegible]
RESEARCH OUTCOME TOOLS
